# Gene–Environment Interaction on Type 2 Diabetes Risk among Chinese Adults Born in Early 1960s

**DOI:** 10.3390/genes13040645

**Published:** 2022-04-05

**Authors:** Chao Song, Weiyan Gong, Caicui Ding, Rui Wang, Hongyun Fang, Ailing Liu

**Affiliations:** National Institute for Nutrition and Health, Chinese Center for Disease Control and Prevention, Beijing 100050, China; songchao@ninh.chinacdc.cn (C.S.); gongwy@ninh.chinacdc.cn (W.G.); dingcc@ninh.chinacdc.cn (C.D.); wangrui@ninh.chinacdc.cn (R.W.); fanghy@ninh.chinacdc.cn (H.F.)

**Keywords:** SNPs, T2D, gene–environment interaction

## Abstract

Background: Gene–environment interactions on type 2 diabetes (T2D) risk are studied little among Chinese adults. Aim: This study aimed to explore the interactions among Chinese adults born in early 1960s. Methods: The interaction of single nucleotide polymorphisms (SNPs) and environmental factors on T2D risk were analyzed by multiple linear or logistic regression models, and in total 2216 subjects were included with the age of 49.7 ± 1.5 years. Results: High dietary intake increased the effects of rs340874 on impaired fasting glucose (IFG), rs5015480, rs7612463 on T2D (OR = 2.27, 2.37, 11.37, respectively), and reduced the effects of rs7172432 on IFG, rs459193 on impaired glucose tolerance (IGT) (OR = 0.08, 0.28, respectively). The associations between rs4607517 and T2D, rs10906115 and IGT, rs4607103, rs5015480 and IFG could be modified by drinking/smoking (OR = 2.28, 0.20, 3.27, 2.58, respectively). Physical activity (PA) interacted with rs12970134, rs2191349, rs4607517 on T2D (OR = 0.39, 3.50, 2.35, respectively), rs2796441 and rs4607517 on IGT (OR = 0.42, 0.33, respectively), and rs4430796, rs5215, and rs972283 on IFG (OR = 0.39, 3.05, 7.96, respectively). Significant interactions were identified between socioeconomic status and rs10830963, rs13266634 on T2D (OR = 0.41, 0.44, respectively), rs1470579 and rs2796441 on IGT (OR = 2.13, 2.37, respectively), and rs7202877 and rs7612463 on IFG (OR = 5.64, 9.18, respectively). Conclusion: There indeed existed interactions between environmental factors and genetic variants on T2D risk among Chinese adults.

## 1. Introduction

Diabetes is a major health issue that has reached alarming levels. The data from the WHO show that about 422 million people worldwide have diabetes, the majority living in low- and middle-income countries [1]. Previous studies reported that the global prevalence of diabetes among adults aged 20 to 79 years was 9.3% (463 million people) in 2019. Moreover, this prevalence is anticipated to reach 10.2% (578 million) in 2030 and an astonishing 10.9% (700 million) in 2045 [2,3]. The latest epidemiological study suggested that 10.9% of Chinese adults had diabetes, with a significant proportion remaining undiagnosed in 2013, and the proportion increased to 12.4% in 2018 [1,4,5,6].

The most common in diabetes is type 2 diabetes (T2D), which accounts for approximately 90% of the total [3]. The past few decades witnessed the dramatical rise of T2D in countries with all income levels, and the global T2D age-standardized incidence and disability-adjusted life year rates also increased, especially in southeast Asia [2,3,5]. T2D is a multifactorial, complex disease resulting from the interplay of genetic, environmental, and epigenetic factors. Multiple established factors have been reported to contribute to the dramatic rise in prevalence of T2D, such as inadequate food intake, low PA, obesity, etc. [2,5,6,7,8,9,10]. Additionally, the impact of T2D differs by population, depending on some variables (e.g., age, race, ethnicity, geography, and socioeconomic status) [11]. Previous studies have found that some environmental factors may regulate the expression of genes and influence susceptibility of T2D, such as gene–diet interaction, in smokers and alcohol drinkers [11,12,13,14,15,16,17]. Few studies have examined the interacitons of other environmental factors with single nucleotide polymorphisms (SNPs) on T2D risk in the Chinese population. Thus, we used the data from the China National Nutrition and Health Survey (CNNHS) 2010–2012 to explore whether there existed gene–environment interactions on T2D risk among Chinese adults born in the early 1960s.

## 2. Materials and Methods

### 2.1. Data Resources

This study was based on 2010–2012 China National Nutrition and Health Surveillance (CNNHS). The CNNHS 2010–2012 was a national representative cross-sectional study covering all 31 provinces, autonomous regions, and municipalities throughout China (except for Taiwan, Hong Kong, and Macao). The survey was conducted using a stratified multistage cluster random sampling method which has been described in a previous study [18]. In total, 2216 subjects born in 1960, 1961, and 1963 were selected into the current study. The exclusion criteria were: unqualified blood sample; failure in DNA extraction; abnormal genetic detecting results; incomplete basic information; the subjects suffered with liver/kidney/heart disease/cancer; subjects diagnosed with T2D and had changed their lifestyle. Questionnaires were used to collect information on the demographic characteristics, dietary and PA behaviors, and health status. Height was measured using a stadiometer after removing shoes, body weight was measured with light clothes using a beam scale. Blood samples were also collected from the subjects.

The protocols of the 2010–2012 CNNHS and “Fetal origin hypothesis of diabetes: thrifty genotype hypothesis or thrifty phenotype” were both approved by the Ethical Committee of the National Institute for Nutrition and Health, Chinese Center for Disease Control and Prevention (2013-018,2013-010). Signed consent forms were obtained from all subjects.

### 2.2. Assessments of Variables

The data included basic household information, individual dietary behaviors (including 24 h dietary inquiring survey for 3 consecutive days and weighing of household seasonings), PA behaviors, individual health status. Environmental factors for the current study included economic level, education level, physical exercise, leisure sedentary behavior, meat and poultry intake, the intake of cereals and beans, smoking, and drinking.

The Chinese Dietary Guideline recommends the reference intake of meat and poultry to be between 40 and 75 g, and the intake of cereals and beans between 50 and 150 g [19]. Thus, we assessed the two variables as follows: the intake of meat and poultry was divided into three categories: low (<40 g/d), medium (from ≥40 to ≤75 g/d), and high (>75 g/d), and the intake of cereals and beans was divided into three categories: insufficient (<50 g/d), sufficient (from ≥50 to ≤150 g/d), and excessive (>150 g/d). Body mass index (BMI) was calculated as weight in kilograms divided by height in meters squared (kg/m^2^).

Fasting glucose was measured by collecting morning fasting venous blood samples, and 2 h plasma glucose was collected 2 h after the subjects took 75 g oral glucose. We used criteria proposed by the World Health Organization, International Diabetes Federation, and the American Diabetes Association on diabetes mellitus [20,21,22]. Impaired fasting glucose (IFG) was defined as fasting plasma glucose (FPG) ≥6.1 and <7.0 mmol/L, 2 h plasma glucose <7.8 mmol/L. Impaired glucose tolerance (IGT) was defined as FPG < 7.0 mmol/L and 2 h plasma glucose ≥7.8 and <11.1 mmol/L. T2D was defined as FPG ≥ 7.0 mmol/L and/or 2 h plasma glucose ≥11.0 mmol/L and/or a previous clinical diagnosis of T2D. Fasting serum insulin (FINS) was measured by Iodine [^125^I] Insulin Radioimmunoassay Kit (Beijing North Institute of Biotechnology Co., Ltd., Beijing, China).

### 2.3. Genotyping

A mass array system (Agena, San Diego, CA, USA) was used to detect the genotypes of 61 SNPs. At the individual level, we removed the samples whose call rates were less than 50%. At the SNP level, we excluded the SNPs if their call rate was <80% and/or their *p*-value for HWE was <0.0001 in subjects without T2D. Finally, 2216 subjects and 50 SNPs were included in the analysis.

### 2.4. Statistical Analysis

The statistical software package SAS version 9.4 (SAS Institute, Cary, NC, USA) was used for data analysis. Two-tailed *p* < 0.05 was considered significant. Firstly, exploratory factor analysis was used to classify the environmental factors. Secondly, the factors with eigenvalue > 1 and cumulative contribution rate > 70% were selected as the initial common factors. The variables with factor load ≥ 0.50 were considered as the main component of the factor after orthogonal rotation. Finally, according to the 50th percentile of the factor score, each factor was divided into two categories of variables. Interactions were tested by creating interaction terms for each genetic variant (coded 0, 1 for carrying the risk allele). We tested the multiplicative interaction with the environmental factors with and without the cross-product term. General linear model regression was used to test the relationship between FPG, FINS, and SNPs, adjusting for age and gender. Logistic regression was used to estimate the ORs for the risk of T2D, IFG, and IGT after adjusting for age and gender.

## 3. Results

### 3.1. Subjects’ Characteristics

A total of 2216 subjects were included in the current study, with the age of 49.7 ± 1.5 years. The basic characteristics of study subjects are presented in Table 1. There was a gender difference in the prevalence of diabetes (*p* < 0.05). There was a significant difference in the prevalence of diabetes between the group with or without family history of diabetes (*p* < 0.05). The prevalence of IGT and IFG varied by their physical exercise status (*p* < 0.05). There were significant differences between diabetes and non-diabetes in age, BMI, FPG, and FINS (*p* < 0.05). There were significant differences between IGT, IFG and non-IGT, non-IFG in BMI and FPG (*p* < 0.05).

### 3.2. Results of Exploratory Factor Analysis

Explorative factor analysis was conducted for eight variables, including education level, economic level, smoking, drinking, intake of meat and poultry, intake of cereals and beans, physical exercise, and sedentary behavior. The KMO test value was 0.500, and the *p*-value of the Bartlett’s spherical test was <0.05, so exploratory factor analysis was performed. Four factors were extracted. Factor 1 (including intake of meat and poultry, intake of cereals and beans) was defined as dietary intake. Factor 2 (including drinking and smoking) was defined as drinking/smoking. Factor 3 (including physical exercise and sedentary behavior) was defined as PA. Factor 4 (including education level and economic level) was defined as socioeeconomic status(Table 2).

### 3.3. Interactions of Gene–Environment on FPG and FINS

As shown in Table 3, high dietary intake could reduce the effect of rs459193 on FPG (β = −0.293 mmol/L, *p* = 0.019), increase the effect of rs7612463 on FPG (β = 0.661 mmol/L, *p* = 0.013). High socioeconomic status decreased the effect of rs13266634, rs2028299, and rs780094 and increased the effect of rs4607517 on FPG (*p* < 0.05). High dietary intake increased the effect of rs10830963, rs10946398, rs11634397, rs12454712, rs13266634, rs1535500, and rs7041847 on FINS (*p* < 0.05). Drinking/smoking could reduce the effect of rs459193 on FINS (β = −2.098 mU/L, *p* = 0.023). PA could reduce the effect of rs12970134 and rs4607103 on FINS (*p* < 0.05).

### 3.4. Interactions of Gene–Environment on Diabetes, IGT, IFG

Table 4 shows the interactions between genetic variants and environment factors on T2D risk. rs5015480 and rs7612463 were found to interact with dietary intake on T2D (OR = 2.37, 11.37, respectively), and rs459193 was found to interact with dietary intake on IGT (OR = 0.28, 95% CI: 0.11–0.72). Significant interaction was found between dietary intake and rs340874 and rs7172432 on IFG (OR = 2.27, 0.08, respectively).

Interaction on T2D was observed between rs4607517 and drinking/smoking (OR = 2.28, 95% CI: 1.02–5.11). Interaction on IGT was observed between rs10906115 and drinking/smoking (OR = 0.20,95% CI: 0.06–0.66), interaction on IFG between rs4607103, rs5015480 and drinking/smoking (OR = 3.27, 2.58, respectively).

PA interacted with rs12970134, rs2191349, and rs4607517on T2D (OR = 0.39, 3.50, 2.35, respectively), rs2796441 and rs4607517 on IGT (OR = 0.42, 0.33, respectively), rs4430796, rs5215, and rs972283 on IFG (OR = 0.39, 3.05, 7.96, respectively).

A significant interaction was also identified between socioeconomic status and rs10830963 and rs13266634 on T2D (OR = 0.41, OR = 0.44, respectively), rs1470579 and rs2796441 on IGT (OR = 2.13, OR = 2.37, respectively), rs7202877 and rs7612463 on IFG (OR = 5.64, OR = 9.18, respectively).

## 4. Discussion

The present study identified several environmental factors that could influence the effects of 25 SNPs on T2D risk indicators (FPG, FINS, T2D, IGT, and IFG), including dietary intake, drinking/smoking, PA, and socioeconomic status.

Previous studies have explored some dietary factors that may affect the associations between some SNPs (rs4607517, rs10830963) and T2D or gestational diabetes (GDM), such as sweet consumption, hypocaloric diet, sugar-sweetened beverages, coffee consumption, etc., and provide novel insights for the prevention and assessment of T2D or GDM [23,24,25]. A case-control study of Chinese women identified the interaction between GCK-rs4607517 and sweets consumption on GDM [23]. In the current study, we examined the interaction between rs4607517 and environmental factors on T2D risk but did not find significant results between rs4607517 and the dietary factors [23]. Maybe we could take the other dietary factors associated with T2D into our consideration in a future study, such as fruits, vegetables, sweet, daily energy, and so on. A randomized dietary intervention trial detected a relationship between rs10830963 and changes of insulin resistance modification induced by two different hypocaloric interventions for nine months, which suggested that genetic risk could be modified by environmental factors including nutrients [25,26]. A significant interaction was also observed between the rs10830963 genotypes and the lifestyle intervention on age-adjusted occurrence of GDM in southern Finland women [27]. Sugar-sweetened beverages were reported to interact with the G/G genotype of rs10830963 in the Chilean population [28]. Our results also found that the association between rs10830963 and FINS could be modified by dietary factors, moreover, we detected that socioeconomic status could interact with the association between this SNP and T2D. A pooled analysis of four Korean prospective studies examined whether the incidence of T2D was related to the consumption of coffee and whether this relationship was modified by some SNPs (including rs5215) related to T2D but did not observe significant interactions [24].

Our data found significant interactions between rs10906115, rs459193, rs4607103, rs4607517, rs5015480 and drinking/smoking, and showed that drinking/smoking decreased the association between rs10906115 and IGT, between rs459193 and FINS, increased the association between rs4607103 and IFG, between rs4607517 and T2D, and between rs5015480 and IFG. A study conducted in Korea found that genetic risk score (GRS, calculated by four SNPs including HHEX-rs5015480) interacted with alcohol intake and increased the risk of development of T2D in the subjects with higher homeostasis model assessment (HOMA-B, an index of insulin secretion capacity) [29].The joint effect of smoking on the association of diabetes with the rs5015480 polymorphism among Korean subjects was examined, which was in line with our findings that rs5015480 interacted with drinking/smoking on T2D risk [30]. However, we did not consider drinking and smoking separately, and additionally, we did not collect specific quantitative indicators of drinking and smoking. Wang et al. found synergistic interactions between rs780094, smoking, and alcohol drinking on hyper-triglyceride waist (HTGW), which is a specific metabolic abnormality associated with T2D, in men in the Henan province of China [31]. Although we also examined the interaction between this SNP and drinking/smoking on T2D risk, we did not find significant results. A future study could be done to detect and verify the interactions we found.

The effect varied depending on different SNPs and PA on T2D. Our data suggested that higher PA could reduce the effects of rs12970134 on FINS and T2D, rs2796441 and rs4607517 on IGT, rs4430796 on IFG, rs4607103 on FINS, increase the effects of rs2191349 and rs4607517on T2D, rs5215 and rs972283 on IFG. Many previous studies have shown that regular PA contributes to the prevention and management of T2D, however, few studies examined the interaction of PA, genes, and T2D risk [10,16,32]. Wang et al. found an antagonistic interaction between rs780094 and severe activity in both men and women on HTGW, [31]. However, our results only found the antagonistic interaction between rs780094 and socioeconomic status on FPG and did not find the interactions between this SNP and other behavior factors. A study which was also conducted in the Henan province of China examined the interaction between PA level with rs12104705 on T2D and found that moderate and high PA with the C-C genotype was associated with decreased risk of T2D as compared with that in low PA with the genotype [15]. Although we examined different T2D-related SNPs, it still suggested that the interaction indeed existed on the occurrence of T2D, and further study in a larger sample needs to be done to confirm the conjecture of these findings.

It is worth mentioning that some research has also reported that several SNPs may interact with some behavior factors on obesity risk, such as rs12970134 and rs12454712 with PA, and rs12970134 and rs13266634 with dietary behaviors [33,34]. Thus, we deduced that maybe the interactions between these SNPs and obesity indicators led to the significant interactions on T2D, as obesity is a risk factor of T2D.

Lifestyle interventions play a crucial role in the prevention and management of T2D, and our results also showed that dietary intake, drinking/smoking, PA, and socioeconomic status could modify the association between variants and T2D. Further study can focus on specific points, such as dietary intake, alcohol and tobacco intake, different types or intensity of PA, and socioeconomic status.

## 5. Conclusions

Engaging in physical exercise, higher dietary intake, drinking/smoking, and higher socioeconomic status may increase or decrease the effects depending on different SNPs. Our results suggested that there existed interactions between environment factors and genetic variants on T2D risk. To our knowledge, it was the first attempt to examine the interactions of so many SNPs and environment factors on different outcomes of T2D, and we did find that some variants interacted with environmental factors on T2D. There are still some limitations. Although we considered the dietary factors, only the intake of cereals and beans and the intake of meat and poultry were analyzed in the study, although other dietary factors may also contribute to the occurrence of T2D. More environmental factors could possibly be analyzed in the future, such as other dietary factors or dietary patterns, physical activities, sleep, etc. Additionally, the variants need to be confirmed and replicated in larger populations, other adult age groups or other ethnic populations.

## Figures and Tables

**Table 1:** Basic characteristics of the subjects.

Variables	Total	Diabetes	IGT	IFG
Total	2216	137 (6.2%)	119 (5.7%)	129 (6.2%)
Age (year)	49.7 ± 1.5	50.2 (49.0,51.6) *	49.7 (48.7,51.6)	49.8 (48.7,51.2)
Gender (*n*,%)				
Male	879 (39.7%)	68 (7.7%) *	38 (4.7%)	43 (5.3%)
Female	1337 (60.3%)	69 (5.2%)	81 (6.4%)	86 (6.8%)
Education Level (*n*,%)				
Illiterate to primary school	787 (35.5%)	48 (6.1%)	38 (5.1%)	45 (6.1%)
Junior middle school	951 (42.9%)	49 (5.2%)	53 (5.9%)	56 (6.2%)
Senior high school or higher	478 (21.6%)	40 (8.4%)	28 (6.4%)	28 (6.4%)
Family’s economic level (Yuan/year/per capita) (*n*,%)				
<20,000	1146 (51.7%)	76 (6.6%)	61 (5.7%)	66 (6.2%)
20,000–40,000	834 (37.6%)	46 (5.5%)	44 (5.6%)	48 (6.1%)
>40,000	157 (7.1%)	8 (5.1%)	9 (6.0%)	9 (6.0%)
Unknown	79 (3.6%)	7 (8.9%)	5 (6.9%)	6 (8.3%)
Smoking (*n*,%)				
No	1555 (70.2%)	86 (5.5%)	89 (6.1%)	95 (6.5%)
Yes	658 (29.7%)	51 (7.8)	30 (4.9%)	34 (5.6%)
Unknown	3 (0.1%)	0	0	0
Drinking (*n*,%)				
No	1472 (66.4%)	87 (5.9%)	82 (5.9%)	97 (7.0%)
Yes	742 (33.5%)	50 (6.7%)	37 (5.4%)	32 (4.6%)
Unknown	2 (0.1%)	0	0	0
Intake of meat and poultry (*n*,%)				
Low	692 (31.2%)	39 (5.6%)	33 (5.1%)	32 (4.9%)
Medium	382 (17.2%)	19 (5.0%)	24 (6.6%)	28 (7.7%)
High	605 (27.3%)	36 (6.0%)	37 (6.5%)	37 (6.5%)
Unknown	537 (24.2%)	43 (8.0%)	25 (5.1%)	32 (6.5%)
Intake of cereals and beans (*n*,%)				
Insufficient	1452 (65.5%)	79 (5.4%)	79 (5.8%)	83 (6.1%)
Sufficient	185 (8.3%)	13 (7.0%)	11 (6.4%)	13 (7.6%)
Excessive	42 (1.9%)	2 (4.8%)	4 (10.0%)	1 (2.5%)
Unknown	537 (24.2%)	43 (8.0%)	25 (5.1%)	32 (6.5%)
Physical exercise (*n*,%)				
No	2009 (90.7%)	121 (6.0%)	101 (5.4%) *	111 (5.9%)*
Yes	192 (8.7%)	16 (8.3%)	18 (10.2%)	15 (8.5%)
Unknown	15 (0.7%)	0	0	3 (20.0%)
Sedentary behavior (*n*,%)				
≥2 h/d	349 (15.8%)	22 (6.3%)	18 (5.5%)	21 (6.4%)
<2 h/d	1808 (81.6%)	112 (6.2%)	101 (6.0%)	105 (6.2%)
Unknown	59 (2.7%)	3 (5.1%)	0	3 (5.4%)
Family history of diabetes (*n*,%)				
No	2173 (98.1%)	130 (6.0%) *	115 (5.6%)	125 (6.1%)
Yes	43 (1.9%)	7 (16.3%)	4 (11.1%)	4 (11.1%)
BMI (kg/m^2^)	24.3 ± 3.4	26.1 (23.7,28.4) *	24.8 (22.8,27.7) *	24.5 (22.4,27.1) *
FPG (mmol/L)	5.2 (4.7,5.7)	7.6 (7.0,8.6) *	5.7 (5.2,6.2) *	6.4 (6.3,6.6) *
FINS (mU/L)	12.6 (9.3,15.7)	14.4 (11.2,19.0) *	12.1 (8.8,14.8)	14.2 (9.9,18.1)

* *p* < 0.05.

**Table 2:** Factor analysis of T2D-risk-related environmental factors.

Environmental Factors	Factor 1 *	Factor 2 *	Factor 3 *	Factor 4 *
Intake of cereals and beans	0.998			
Intake of meat and poultry	0.998			
Drinking		0.953		
Smoke		0.952		
Physical exercise			0.867	
Sedentary behavior			0.864	
Education level				0.745
Economic level				0.703
Eigenvalues	2.015	1.814	1.503	1.046
Contribution rate (%)	0.252	0.227	0.188	0.131
Cumulative contribution rate of variance (%)	0.252	0.479	0.667	0.797

* Only displayed the value of factor loading > 0.50, which was considered as the principal component of the factor.

**Table 3:** Interactions of gene–environment on FPG and FINS.

SNP	Dietary Intake		Drinking/Smoke		Physical Activity		Socioeconomic Status	
	β	*p*	β	*p*	β	*p*	β	*p*
FPG								
rs10401969	0.023	0.872	−0.214	0.139	−0.117	0.420	−0.045	0.754
rs10830963	0.167	0.142	0.048	0.670	−0.015	0.897	−0.090	0.430
rs10842994	−0.024	0.940	0.322	0.317	−0.106	0.743	0.133	0.681
rs10886471	−0.097	0.721	0.134	0.622	−0.072	0.796	0.004	0.989
rs10906115	0.052	0.750	0.029	0.860	−0.237	0.143	−0.288	0.075
rs10946398	0.016	0.885	−0.045	0.693	0.051	0.656	0.077	0.498
rs11257655	−0.001	0.995	−0.034	0.813	−0.097	0.498	−0.224	0.119
rs11634397	0.121	0.380	0.123	0.374	0.071	0.608	0.075	0.587
rs12454712	0.096	0.486	−0.015	0.910	0.224	0.102	0.070	0.607
rs12970134	−0.003	0.976	0.013	0.908	−0.034	0.771	0.037	0.750
rs13266634	0.149	0.198	−0.118	0.305	−0.115	0.317	−0.246	0.033 *
rs1470579	0.014	0.897	−0.012	0.910	0.050	0.638	0.073	0.495
rs1535500	0.229	0.051	−0.122	0.300	−0.021	0.858	−0.180	0.126
rs1552224	−0.112	0.875	−0.226	0.752	−0.734	0.311	0.266	0.713
rs1558902	−0.072	0.580	0.092	0.480	0.156	0.229	0.035	0.787
rs16861329	−0.107	0.327	0.070	0.523	0.003	0.981	0.036	0.745
rs17584499	0.024	0.857	−0.131	0.326	−0.062	0.645	−0.033	0.804
rs2028299	0.066	0.545	−0.053	0.628	−0.163	0.138	−0.221	0.044 *
rs2191349	−0.115	0.471	−0.075	0.641	−0.004	0.979	0.129	0.416
rs243021	0.174	0.323	0.079	0.657	−0.250	0.156	0.004	0.983
rs2796441	0.034	0.763	0.121	0.284	−0.018	0.873	0.031	0.786
rs2943641	0.107	0.893	0.302	0.645	−0.002	0.998	0.052	0.939
rs340874	0.085	0.454	0.063	0.575	0.111	0.326	−0.073	0.520
rs3794991	−0.111	0.510	−0.109	0.518	0.056	0.739	−0.015	0.930
rs3923113	−0.385	0.283	0.085	0.815	0.013	0.970	0.048	0.893
rs4430796	0.026	0.807	−0.045	0.680	−0.084	0.440	−0.121	0.266
rs459193	−0.293	0.019 *	0.018	0.886	0.043	0.729	0.210	0.095
rs4607103	−0.012	0.934	0.139	0.357	−0.177	0.242	−0.017	0.909
rs4607517	−0.175	0.115	0.189	0.089	0.095	0.389	0.247	0.026*
rs4858889	0.316	0.418	−0.307	0.431	−0.145	0.706	−0.397	0.306
rs5015480	0.103	0.363	−0.051	0.653	−0.033	0.770	−0.093	0.408
rs516946	−0.109	0.838	0.517	0.335	0.272	0.621	0.158	0.768
rs5215	−0.014	0.903	0.071	0.521	0.148	0.182	−0.056	0.612
rs6815464	0.135	0.359	0.065	0.658	−0.034	0.815	−0.099	0.501
rs7041847	0.063	0.603	0.001	0.993	−0.000	0.997	0.030	0.806
rs7172432	−0.082	0.609	−0.088	0.581	−0.267	0.094	−0.127	0.425
rs7178572	−0.034	0.757	−0.092	0.399	−0.035	0.751	−0.031	0.776
rs7202877	0.050	0.866	0.362	0.227	−0.089	0.769	0.314	0.297
rs7403531	0.104	0.361	−0.070	0.538	0.062	0.588	0.039	0.735
rs7593730	0.196	0.534	−0.136	0.666	−0.319	0.319	−0.199	0.532
rs7612463	0.661	0.013 *	0.009	0.972	0.370	0.157	−0.055	0.834
rs780094	0.104	0.392	−0.124	0.306	−0.081	0.505	−0.253	0.037*
rs7961581	0.096	0.376	0.053	0.622	−0.102	0.346	0.023	0.830
rs8050136	−0.041	0.745	0.114	0.367	0.132	0.298	0.023	0.856
rs8090011	−0.368	0.070	−0.062	0.758	0.353	0.083	0.177	0.384
rs831571	0.137	0.206	−0.134	0.217	−0.134	0.216	−0.210	0.052
rs864745	0.073	0.775	−0.324	0.205	−0.078	0.757	−0.072	0.778
rs896854	−0.111	0.299	0.142	0.182	0.191	0.073	0.062	0.560
rs9470794	0.027	0.877	0.064	0.717	0.075	0.670	0.257	0.143
rs972283	0.216	0.285	−0.293	0.149	0.015	0.942	−0.272	0.179
FINS								
rs10401969	−0.954	0.373	1.842	0.085	0.747	0.486	1.425	0.183
rs10830963	1.917	0.022*	0.025	0.976	1.495	0.075	−0.953	0.256
rs10842994	0.808	0.726	1.274	0.580	0.048	0.983	−1.223	0.596
rs10886471	−0.810	0.674	3.352	0.074	0.801	0.677	1.394	0.462
rs10906115	1.269	0.286	−0.327	0.785	0.711	0.549	0.173	0.884
rs10946398	1.860	0.026 *	−0.307	0.713	0.609	0.466	−0.386	0.643
rs11257655	1.607	0.121	−0.340	0.743	0.316	0.760	−0.916	0.376
rs11634397	2.290	0.026 *	0.650	0.529	−0.300	0.771	−1.351	0.190
rs12454712	2.394	0.017 *	−0.287	0.775	1.352	0.177	−1.187	0.236
rs12970134	−0.447	0.595	−1.336	0.112	−1.842	0.028*	−1.435	0.088
rs13266634	2.137	0.010 *	−0.818	0.327	1.173	0.160	−0.806	0.334
rs1470579	1.218	0.121	0.071	0.928	0.018	0.981	−0.444	0.572
rs1535500	1.760	0.043 *	−0.339	0.697	0.791	0.363	−0.659	0.449
rs1552224	−2.874	0.563	−0.001	1.000	−3.856	0.477	1.909	0.706
rs1558902	−0.236	0.801	0.051	0.956	0.725	0.440	−0.001	0.999
rs16861329	0.144	0.857	−0.403	0.614	−1.014	0.204	−0.391	0.624
rs17584499	0.674	0.494	0.922	0.349	0.249	0.800	−0.130	0.895
rs2028299	0.981	0.218	−0.918	0.248	−0.435	0.585	−0.252	0.752
rs2191349	−0.112	0.922	0.389	0.733	0.520	0.645	−0.036	0.975
rs243021	−0.899	0.484	0.893	0.491	−0.433	0.736	1.298	0.313
rs2796441	0.126	0.878	−0.167	0.838	0.632	0.439	0.298	0.716
rs2943641	0.172	0.974	0.707	0.882	−1.820	0.711	−2.770	0.559
rs340874	1.015	0.197	0.608	0.440	1.189	0.131	0.776	0.324
rs3794991	−0.432	0.726	0.953	0.440	−0.710	0.567	0.441	0.721
rs3923113	4.480	0.143	−2.103	0.503	0.804	0.793	−2.256	0.460
rs4430796	−1.448	0.062	−1.513	0.051	−0.947	0.222	−0.383	0.622
rs459193	−1.028	0.266	−2.098	0.023 *	−1.388	0.133	−1.547	0.094
rs4607103	−0.502	0.652	0.038	0.973	−2.265	0.042 *	0.111	0.921
rs4607517	−0.131	0.870	0.115	0.886	0.084	0.917	0.096	0.904
rs4858889	−1.917	0.489	1.132	0.682	−2.236	0.414	0.836	0.761
rs5015480	0.579	0.481	−1.064	0.196	−1.220	0.138	−1.103	0.180
rs516946	−1.652	0.719	−0.384	0.932	−1.005	0.834	−0.707	0.878
rs5215	0.176	0.829	−1.260	0.121	−0.757	0.351	−1.320	0.104
rs6815464	0.424	0.695	−0.939	0.387	0.064	0.953	−0.937	0.387
rs7041847	1.781	0.043 *	−0.162	0.854	0.058	0.947	−0.388	0.660
rs7172432	−0.381	0.739	0.383	0.739	−1.925	0.093	0.070	0.951
rs7178572	0.085	0.915	0.417	0.600	−0.529	0.506	−0.460	0.563
rs7202877	−2.652	0.223	1.633	0.448	−1.356	0.539	2.799	0.194
rs7403531	0.226	0.788	0.576	0.493	0.174	0.836	0.081	0.923
rs7593730	0.931	0.720	−0.824	0.758	1.128	0.683	0.757	0.785
rs7612463	0.826	0.666	0.288	0.882	−1.878	0.325	−1.528	0.424
rs780094	1.516	0.091	−0.601	0.503	0.105	0.907	−1.446	0.107
rs7961581	−1.140	0.146	0.139	0.859	−0.451	0.565	0.793	0.313
rs8050136	−0.549	0.555	0.789	0.396	0.116	0.901	0.335	0.719
rs8090011	−1.163	0.440	2.631	0.080	2.296	0.130	2.529	0.092
rs831571	0.878	0.269	0.510	0.521	0.030	0.970	0.019	0.981
rs864745	1.915	0.326	−1.338	0.500	2.279	0.239	−1.862	0.343
rs896854	0.383	0.624	0.306	0.696	1.224	0.117	0.163	0.835
rs9470794	0.051	0.968	−0.253	0.842	0.301	0.813	0.732	0.564
rs972283	−1.306	0.378	0.404	0.785	−0.187	0.899	1.155	0.435

* *p* < 0.05.

**Table 4:** The interaction between each factor and SNPs on T2D risk.

SNP	Dietary Intake			Drinking/Smoke			Physical Activity			Socioeconomic Status		
	Diabetes	IGT	*IFG*	Diabetes	IGT	*IFG*	Diabetes	IGT	*IFG*	Diabetes	IGT	*IFG*
rs10401969	0.909	0.774	0.928	0.291	0.334	0.108	0.832	0.839	0.915	0.524	0.727	0.911
rs10830963	0.992	0.141	0.678	0.225	0.893	0.085	0.306	0.944	0.898	0.022 *	0.199	0.922
rs10842994	0.695	0.367	0.867	0.517	0.522	0.945	0.951	0.613	0.994	0.497	0.844	0.270
rs10886471	0.982	0.949	0.647	0.273	0.938	0.562	0.144	0.954	0.944	0.910	0.951	0.933
rs10906115	0.975	0.167	0.732	0.780	0.008 *	0.520	0.283	0.188	0.836	0.081	0.271	0.649
rs10946398	0.977	0.474	0.764	0.734	0.385	0.500	0.169	0.563	0.269	0.935	0.247	0.812
rs11257655	0.600	0.463	0.964	0.234	0.107	0.236	0.325	0.231	0.440	0.089	0.914	0.403
rs11634397	0.243	0.171	0.736	0.840	0.167	0.314	0.322	0.551	0.992	0.520	0.171	0.812
rs12454712	0.866	0.635	0.865	0.755	0.443	0.489	0.332	0.914	0.096	0.546	0.903	0.622
rs12970134	0.137	0.436	0.325	0.131	0.892	0.433	0.032 *	0.781	0.694	0.156	0.528	0.848
rs13266634	0.211	0.695	0.579	0.161	0.607	0.070	0.395	0.423	0.079	0.028 *	0.602	0.494
rs1470579	0.259	0.157	0.908	0.491	0.975	0.579	0.978	0.415	0.655	0.473	0.049 *	0.726
rs1535500	0.413	0.432	0.250	0.606	0.106	0.106	0.812	0.944	0.513	0.566	0.474	0.765
rs1552224	0.945	0.952	0.941	0.943	0.943	0.943	0.953	0.957	0.939	0.957	0.955	0.956
rs1558902	0.236	0.766	0.265	0.463	0.728	0.800	0.458	0.427	0.297	0.057	0.866	0.328
rs16861329	0.300	0.933	0.399	0.639	0.705	0.689	0.361	0.481	0.716	0.279	0.982	0.613
rs17584499	0.873	0.794	0.514	0.529	0.272	0.772	0.645	0.940	0.156	0.152	0.294	0.571
rs2028299	0.316	0.465	0.098	0.741	0.195	0.897	0.089	0.597	0.537	0.380	0.630	0.115
rs2191349	0.814	0.659	0.599	0.831	0.149	0.499	0.029 *	0.579	0.711	0.354	0.758	0.643
rs243021	0.207	0.335	0.634	0.434	0.308	0.976	0.157	0.135	0.251	0.299	0.443	0.579
rs2796441	0.268	0.189	0.275	0.235	0.727	0.502	0.608	0.041 *	0.251	0.805	0.044 *	0.600
rs2943641	0.999	0.963	0.959	0.999	0.937	0.991	1.000	0.960	0.828	0.999	0.957	0.945
rs340874	0.670	0.749	0.033 *	0.723	0.161	0.968	0.399	0.777	0.939	0.941	0.537	0.435
rs3794991	0.118	0.593	0.759	0.748	0.702	0.387	0.447	0.660	0.914	0.528	0.728	0.677
rs3923113	0.982	0.950	0.944	0.871	0.934	0.557	0.951	0.880	0.822	0.990	0.956	0.950
rs4430796	0.354	0.963	0.866	0.925	0.788	0.407	0.842	0.405	0.014 *	0.510	0.597	0.977
rs459193	0.467	0.008 *	0.280	0.240	0.072	0.345	0.522	0.750	0.800	0.381	0.141	0.602
rs4607103	0.504	0.506	0.999	0.447	0.646	0.023 *	0.063	0.290	0.335	0.639	0.594	0.145
rs4607517	0.865	0.961	0.598	0.045 *	0.091	0.423	0.038 *	0.010 *	0.374	0.104	0.290	0.127
rs4858889	0.667	0.943	0.155	0.962	0.939	0.748	0.879	0.937	0.885	0.239	0.945	0.153
rs5015480	0.036 *	0.443	0.420	0.636	0.420	0.020 *	0.811	0.965	0.175	0.618	0.299	0.786
rs516946	0.953	0.918	0.465	0.896	0.947	0.942	0.879	0.938	0.954	0.849	0.833	0.939
rs5215	0.822	0.292	0.717	0.981	0.206	0.288	0.677	0.691	0.009 *	0.408	0.662	0.056
rs6815464	0.224	0.475	0.479	0.842	0.990	0.766	0.280	0.733	0.218	0.515	0.257	0.298
rs7041847	0.962	0.570	0.553	0.610	0.771	0.426	0.278	0.997	0.711	0.757	0.268	0.128
rs7172432	0.926	0.611	0.014 *	0.732	0.168	0.759	0.377	0.955	0.708	0.744	0.492	0.856
rs7178572	0.942	0.077	0.165	0.507	0.527	0.139	0.930	0.763	0.497	0.349	0.867	0.462
rs7202877	0.947	0.945	0.789	0.952	0.945	0.209	0.958	0.761	0.807	0.951	0.952	0.038 *
rs7403531	0.130	0.493	0.297	0.576	0.945	0.431	0.076	0.115	0.905	0.919	0.368	0.629
rs7593730	0.905	0.390	0.944	0.877	0.301	0.979	0.958	0.462	0.413	0.816	0.716	0.496
rs7612463	0.038 *	0.790	0.673	0.664	0.896	0.207	0.237	0.947	0.615	0.354	0.927	0.038 *
rs780094	0.522	0.416	0.135	0.880	0.913	0.677	0.615	0.708	0.624	0.618	0.112	0.128
rs7961581	0.951	0.518	0.678	0.511	0.348	0.856	0.269	0.516	0.851	0.123	0.057	0.665
rs8050136	0.127	0.230	0.270	0.271	0.963	0.888	0.395	0.659	0.759	0.050	0.343	0.643
rs8090011	0.193	0.819	0.065	0.122	0.163	0.849	0.954	0.866	0.777	0.316	0.387	0.088
rs831571	0.979	0.966	0.079	0.731	0.633	0.312	0.961	0.887	0.191	0.381	0.497	0.372
rs864745	0.105	0.159	0.170	0.240	0.810	0.814	0.935	0.949	0.319	0.128	0.181	0.455
rs896854	0.964	0.915	0.807	0.423	0.950	0.307	0.248	0.305	0.763	0.901	0.570	0.204
rs9470794	0.247	0.595	0.720	0.631	0.169	0.497	0.485	0.723	0.454	0.949	0.469	0.098
rs972283	0.531	0.074	0.441	0.333	0.964	0.237	0.220	0.617	0.039 *	0.371	0.138	0.485

Data are presented as *p*-value; * *p* < 0.05.

## Data Availability

Not applicable.
